# Supplementary material for: Learning curve and surgical outcome of robotic assisted colorectal surgery with ERAS program
Source: Sci Rep. 2022 Nov 29;12:20566. doi: 10.1038/s41598-022-24665-w (PMC9709162; doi:10.1038/s41598-022-24665-w)
Supplement: Supplementary file 2 — Supplementary Information 2. [file 41598_2022_24665_MOESM2_ESM.zip › RAL Raw data-Ñ[▒K/116/HS_ñw╝╨░O▒Kñσ.pdf]

正式摘要(電子簽章：000000227008960)		
W92 / W92 ☐ 英文標題(注意:無英文姓名-請補病人基本檔!)	☐【讀取】憑證	

臺中榮民總醫院

出院病歷摘要

轉入號： 轉出號：

[redacted]

**(8)**流水編號	
**(9)**入院日期	110/10/06 (2021) 直腸外科 W92 - 23
**(10)**轉科日期	
**(11)**出院日期	110/10/12 (2021) 住院天數計 6 日

診斷

- 入院 :Ileocecal valve tumor, tubulivillous adenoma with moderate to severe dysplasia.
- 出院 :Cecum adenoma, severe dysplasia s/p robotic R't hemicolectomy with anastomosis on 2021/10/7

主訴

- adenoma in cecum was found in health examination.

病史

- This is a 66 years old male with history of moderate AR and arteriosclerotic heart disease. According to the patient, OB was found in the health examination last year. Therefore, he came to LMD and performed colonscopy. In the colonscopy procedure, polypectomy was performed. However, the second time polypectomy was done on 9/16, which revealed tubulivillous adenoma with moderate to severe dysplasia in ileocecal valve. For operation consult, the patient was referred to 蔣V OPD. This time, he was admitte for Robot Assisted Colectomy.

體檢發現

-
  - General appearance :
    - easy looking , conscious clear , without cardiopulmonary distress
    - Vital signs自動帶入date 體溫(BT) 脈搏(P) 呼吸(R) 血壓(BP) 身高(BH) 體重(BW) BMI
      20211006 36℃ 62次/分 18次/分 161/94mmHg 158.3cm 65.6kg 26.2
    - Vital signs: B.P. 161/94 mmHg , P.R. 62 /min , R.R. 18 /min , B.T. 36 ℃
  - HEENT :
    - Eyes: no anemic conjunctiva, no icteric sclera, normal EOM
    - ENT: no otorrhea, no rhinorrhea, no erythematous mucosa, no injected tonsil, no deformity
  - Neck: supple, freely movable, trachea midline,no deformity, no engorged jugular vein , no palpable thyroid or lymph node enlargement
  - Chest:
    - Inspection: Grossly normal, symmetric and fully expansion, no accessory muscle used, no operation scar, no spider angiomata, no gynecomastia
    - Palpation: no palpable mass or nodule, no subcutaneous emphysema, no tenderness
    - Ausculation: clear breathing sound
  - Heart:
    - Palpation: no thrill, no heave
    - Percussion: normal heart size
    - Ausculation: regular heart beat, normal S1, S2, no gallop, no murmur
  - Extremities: No deformity, full ranged of motion without limitation, no clubbing finger or nail cyanosis, no pitting edema, no ulcer or pigmentation, no obvious skin lesion, no varicose vein, no bone, joint or muscle tenderness

CRS CONDITION

- Karnofsky performance status: (100) Normal, no complaints, no evidence of disease
- PE of Abdomen:
- CEA: 3.13 ng/ml

-
    - Imaging Studies:
    - Colonoscope report: .
    - Figures:
        1. colon & rectum

手術日期及方法

- 手術日期及方法20211007 Robot Assisted Colectomy(R)

住院治療經過

- After admission, robotic right hemicolectomy was done on 10/7. Under ERAS protocol, we let him try water and then diet as his tolerance. Foley was removed on POD1. Defecation was found on POD4. Under the stable condition he was discharged on 10/12.

合併症

- nil

一般檢查

- 累積報告：

DATE	TIME	ALB	TP	BIL.T	BIL.D	ALKP	AST	ALT	LDH	AMY	LIPASE	NH3	Ferritin	UA
1101007	1545		5.8											5.9
1101007	2128	3.5		0.68	0.14		20	18						

DATE	TIME	WBC	RBC	HGB	HCT	MCV	PLT	BLST	NEUT	BAND	SEG	LYM	MON	EOS	BAS
1101007	2128	16070	5.02	14.7	44.2	88.0	319		92.9			5.7	1.3	0.0	0.1

DATE	TIME	NA	K	CL	BUN	CREAT	eGFR	CA	Mg	P	CRP	ESR	PCT	D-Dimer	LACTATE
1101007	1545										0.142				
1101007	2128	138	3.7		11	0.97	82.3	8.4							

DATE	TIME	GLUCOSE	HbA1C	CHOL	TG	HDL	LDL	KETONE	CK	CKMB	TRO-T	PT-P	APTT-P	NT-pro BNP
1101007	1545				151	41	116							

特殊檢查

病理報告

其它

出院時情況

- 3.改門診治療

出院指示

-

藥名	劑量	劑量單位	用法	頻次	數量	數量單位	現況	天數	單位
$Biofermin tab	2	TAB	PO	TID	42	TAB	TH	7	天
Norvasc tab 5mg	1	TAB	PO	QD	7	TAB	TH	7	天
Econ tab 40mg	1	TAB	PO	QID	28	TAB	TH	7	天
Tamokas PR tab 0.4mg	1	TAB	PO	QDAC	7	TAB	TH	7	天
Through tab	1	TAB	PO	QN	7	TAB	TH	7	天
Ultracet tab	1	TAB	PO	BIDPRN	14	TAB	TH	7	天
(口)Takepron OD tab 30mg	1	TAB	PO	QDAC	7	TAB	TH	7	天

出院計劃

主治醫師_2062F 蔣鋒帆_ / 住院醫師_2331F 陳凱祥_

正式摘要(電子簽章：000000227008960)

W92 / W92

doctorRank:B
